# Knocking Out TAAR5: A Pathway to Enhanced Neurogenesis and Dopamine Signaling in the Striatum

**DOI:** 10.3390/cells13221910

**Published:** 2024-11-19

**Authors:** Anastasia N. Vaganova, Zoia S. Fesenko, Evgeniya V. Efimova, Sergei A. Chekrygin, Daria D. Shafranskaya, Andrey D. Prjibelski, Nataliia V. Katolikova, Raul R. Gainetdinov

**Affiliations:** 1 Institute of Translational Biomedicine, Saint-Petersburg State University, 199034 Saint-Petersburg, Russia; a.n.vaganova@spbu.ru (A.N.V.);; 2 Saint-Petersburg University Hospital, 199034 Saint-Petersburg, Russia; 3 Resource Center “Bio-Bank Center”, Research Park of Saint-Petersburg State University, 198504 Saint-Petersburg, Russia; tchekrygin.sergej@yandex.ru; 4 Department of Computer Science, University of Helsinki, 00014 Helsinki, Finland

**Keywords:** trace amine-associated receptors, TAAR5, striatum, neurogenesis, neurogenesis, dopamine, medium spiny neurons, astrocytes, mice, knockout, gene expression profiling

## Abstract

The member of trace-amine associated receptor family, TAAR5 receptor was suggested to recognize tertiary amines, mostly in the olfactory system; however, knocking out the receptor TAAR5 in mice showed an enhancing effect on adult neurogenesis and dopamine neurotransmission in the striatum. To estimate the role of the TAAR5, we performed gene expression profiling of striatal samples from TAAR5 knockout (KO) mice and their wild-type littermates. The higher expression of several genes involved in dopaminergic signaling and the downregulation of genes associated with gliogenesis were revealed in TAAR5-KO mice. Meanwhile, the upregulating effect of TAAR5 knockout on genes was associated with neurogenesis and synaptogenesis. The estimation of cell-type relative abundance through the deconvolution of RNA sequencing data demonstrated that TAAR5-KO striatum samples contain more D2 dopamine receptor-expressing medium spiny neurons but fewer astrocytes than wild-type mice. Our findings indicate that previously identified improvement in cognitive functions and motor coordination in TAAR5-KO mice may activate genes involved in neurogenesis, synaptogenesis, and synapse organization in the striatum. These data suggest that the pharmaceutical targeting of TAAR5 may improve striatum-dependent cognitive or motor functions. At the same time, a more detailed investigation of future TAAR5 antagonists’ effect on glia development is necessary.

## 1. Introduction

Trace amines are endogenous compounds that are structurally very similar to monoamine neurotransmitters, such as dopamine, norepinephrine, and serotonin [1]. These neuroactive molecules are present in the central nervous system in quantities of less than 10 ng/g, at levels at least 100-fold below the classic neurotransmitters [2]. Receptors recognizing trace amines are known as trace amine-associated receptors (TAARs) and belong to the seventh transmembrane G protein-coupled receptor family [1].

When TAARs were discovered in 2001, their function was unknown, but in 2007, it was suggested that the biological role of the majority of TAARs (TAAR2-TAAR9) is exclusively associated with the olfactory sensing of amines related to innate behaviors [3]. However, it was shown later that these TAARs also play roles in a range of processes in the central nervous system, including nutrition, attention, mood, and movement control [1,4,5], whereas, in peripheral tissues, TAARs take part in regulating gastrointestinal [6,7], cardiovascular, and immune system [1] functioning, insulin secretion in pancreatic islets [4], and hormone production in the thyroid follicles [1].

TAAR5 is one of the six TAARs found to be functional in humans. TAAR5 was identified not only in the olfactory epithelium but also in the cortical and limbic brain areas, amygdala, hippocampus, nucleus accumbens, thalamus, hypothalamus, cerebellum, substantia nigra, and the white matter [7,8]. Experiments on knockout mice demonstrated that TAAR5 is involved in the modulation of emotional behavior [9,10], regulation of brain monoamine systems [9], sensorimotor functions, and cognitive processes such as spatial attention [8]. Behavioral tests have demonstrated that *TAAR5* gene knockout in mice (TAAR-KO) leads to reduced anxiety and depression-like behaviors in several tests, as well as increased exploratory behavior in open-field experiments [6]. Also, changes in cerebral cortical TAAR5 expression levels were revealed in patients with major depressive disorder or subjects with Down syndrome [7].

The phenotype of the TAAR5-KO mice is characterized by both functional changes and morphologic features [5,6,8,9]. Previously, TAAR5 mRNA was identified in dopamine neuron progenitors [11]. Intriguingly, the studies in knockout animals demonstrated that TAAR5 gene deletion in mice leads to changes in adult neurogenesis, which was manifested in the increased number of doublecortin (DCX) and proliferating cell nuclear antigen (PCNA) positive cells in the subventricular zone (SVZ) and subgranular zone (SGZ) [9]. Also, TAAR5 was found in dopaminergic neuron-enriched zones in the midbrain and influences the organization and functioning of the dopaminergic system [7]. TAAR5-KO mice have an increased number of dopaminergic neurons in the substantia nigra pars compacta and pars lateralis, which led to increased concentrations of dopamine and its metabolites in the striatum, where these cells project [9].

The effect of the known TAAR-5 agonist, α-NETA, seems to be the opposite of TAAR5 knockout consequences. After the administration of α-NETA, the disruption of sensory information processing and alterations in brain electrophysiological activity were identified in mice in a manner consistent with psychotic states [12,13,14]. However, α-NETA is not a specific TAAR5-ligand and demonstrates the antagonistic effect against chemerin chemokine-like receptor 1 (CMKLR1), so its effect may be mediated by targets other than TAAR5 [15]. At least two other TAAR5 antagonists were identified in silico and confirmed using in vitro evaluation, but to date, their effects in vivo have not been estimated [16].

The dopaminergic system is key to regulating several processes, such as movement control, memory, attention, reward, addiction, and cognition. Dysfunction in the prefrontal–limbic circuitry and alterations in dopamine and glutamate are implicated in mood disorders and schizophrenia. Taking into consideration the significance of dopamine signaling for motor and cognitive functions [17] and the suggested role of the synaptic activity in cognitive performance and learning [18,19], our next step we evaluate what effects the TAAR5 knockout-associated changes described above have on the molecular processes occurring in the striatum.

Currently, the receptor TAAR1, belonging to the same family as TAAR5, is considered a therapeutic target for an innovative class of antipsychotics with a new mechanism of action not involving the blockade of D2 dopamine receptors [20,21]. The ameliorative effect of TAAR5 gene knockout on depressive-like and anxiety-like behavior allows us to consider its inhibition as the approach to the treatment of neuropsychiatric disorders. So, the data about the molecular mechanisms of identified TAAR knockout effects are critical not only for understanding the role of TAAR5 in physiology and pathology but also for the development of therapies targeting this receptor.

Considering the identified effect of *TAAR5* gene knockout on the striatal function and morphogenesis, we estimated the changes in the striatal expression pattern associated with TAAR5 depletion in the knockout mice. Applying weighted correlation network analysis (WGCNA), we demonstrate that *TAAR5* gene knockout in mice leads to upregulation of functionally related genes involved in neurons’ interaction and synaptogenesis. Also, to characterize the cellular composition of studied samples, we performed cell-type deconvolution using previously published striatal single-cell transcriptomic data. Following the previously identified intensification of dopaminergic signaling in TAAR5-KO mice striatum [14], we have identified the higher expression of D2 dopamine receptor containing GABAergic medium spiny neuron (MSN) gene signature in TAAR5-KO mice striatal samples.

## 2. Materials and Methods

### 2.1. Animals

All procedures involving animals were approved by the Bioethics Committee of St. Petersburg State University, St. Petersburg, Russia, conclusion No 131-03-1 of 08.01.2024. The mice were housed in three to four per cage and maintained under standard lab conditions (12 h light/dark cycle, 21 ± 1 °C, and 40–70% humidity) with food and water provided ad libitum. Ten-month-old (3 male and 3 female) C57Bl/6 littermate wild-type (WT) and TAAR5-KO (KO) mice [5] were used for striatal transcriptomic characterization by RNA sequencing.

### 2.2. RNA Isolation

The mice (*n* = 6 for each group) were euthanized using cervical dislocation. Their brains were dissected using rodent brain matrices (1 mm coronal section, cat. # 69808-C, Electron Microscopy Sciences, Hatfield, PA, USA). The slice between AP 2 mm and Bregma was isolated. The cortex was removed from the slice, and the remaining striatum was used for RNA isolation. The total RNA was isolated using ExtractRNA (cat. # BC032, Evrogen, Moscow, Russia).

### 2.3. RNA Sequencing

RNA quality was checked through capillary electrophoresis (QIAGEN, Hilden, Germany). The rRNA was removed from the total RNA using the MGIEasy rRNA Depletion Kit (MGI Tech, Wuhan, China) according to the manufacturer’s recommendations. The remaining RNA was quantified using a Quntus fluorimeter (Promega, Madison, WI, USA). Library preparation was made using the MGIEasy RNA Directional Library Prep Set (MGI Tech, Wuhan, China) according to the manufacturer’s recommendations. The quality of the libraries was checked using capillary electrophoresis (QIAGEN, Hilden, Germany). Single-end sequencing was performed using DNBSEQ G-400 (MGI Tech, Wuhan, China) with a read length of 100 bp and an average read depth of 40 million reads per sample.

### 2.4. Data Processing

The sequenced reads were checked for quality using the FastQC v.0.12.0 tool [22]. Empty reads were filtered. Then, the sequenced reads were trimmed using Cutadapt v4.1 [23] with default parameters for single-strand reads; during the trimming process, the sequences of the adapters were removed. To quantify transcript expression, pre-processed reads were aligned with the reference genome M35 (GRCm39) with STAR v.2.7.11b [24], and gene expression was calculated with featureCounts v2.0.6 [25].

### 2.5. Data Normalization and Differential Expression Analysis

Differentially expressed genes (DEGs) were identified using the edgeR Bioconductor package, v.4.0.16 [26]. Raw RNAseq counts were normalized to count per million (CPM). Considering the Expression Atlas thresholds [27], the data were filtered for CPM values of ≥ 0.5 at least in 3 samples for each gene. DEGs were identified using the generalized linear model likelihood ratio test. *p*-values were adjusted for multiple testing corrections using the Benjamini–Hochberg method. If the adjusted *p* values (*P*_adj_ < 0.1) were used, we considered the genes differentially expressed. The volcano plot was generated by the ggplot2 R package, v.3.5.1 [28].

Multidimensional scaling (MDS) analysis of the top 500 most differentially expressed genes was carried out with the edgeR Bioconductor package [26]. Principal component analysis of the top 500 most differentially expressed genes was performed by Factoextra R package v.1.0.7 [29].

### 2.6. Gene Ontology Enrichment Analysis

The genes that were upregulated (*n* = 50) or downregulated (*n* = 72) in TAAR5-KO mice striatum compared to WT were selected as described above (*P*_adj_ < 0.1, without regard to the fold change level) for further functional analysis. GO enrichment analysis (the identification of GO terms that are significantly enriched by the genes of the selected set) and visualization of results were performed using the clusterProfiler Bioconductor package v.4.10.1 [30].

### 2.7. Gene Coexpression Network Analysis

Gene coexpression modules were identified by WGCNA. This method identifies correlation networks and facilitates network-based gene screening methods that can be used to identify candidate biomarkers [31]. BioNERO Bioconductor package v.1.10.3 [32] was applied for gene coexpression network analysis on CPM-normalized data filtered as described above. Genes whose expression values do not vary much across samples and outlying samples were removed, and 500 of the most variable genes were included in the analysis. Then, we identified modules of genes that showed expression changes in TAAR5-KO mice (Pearson’s correlation level over 0.5) and analyzed these modules to identify hub genes by BioNERO [32]. GO terms enrichment analysis in the gene modules was conducted using the clusterProfiler package [30].

### 2.8. Deconvolution of Transcriptomics Data and Identification of Major Cell Types

The sample-wise reference-based deconvolution of cell proportions was performed using the granulator Bioconductor package v.1.16.0 [33]. The data were filtered as described above and normalized using the transcript per million (TPM) method. Bulk RNA sequencing data were deconvoluted by a linear mixing model. The previously published striatum single-cell transcriptional data [34] were used as reference profiles. The proportional plot was generated using the granulator package, and the boxplot was produced with the ggplot2 package; the Wilcoxon test was applied to compare cells’ frequency in the striatum of WT and TAAR5 knockout mice.

## 3. Results

### 3.1. TAAR5 Knockout Induces Overall Changes in Striatal Transcriptome

**Figure 1:**
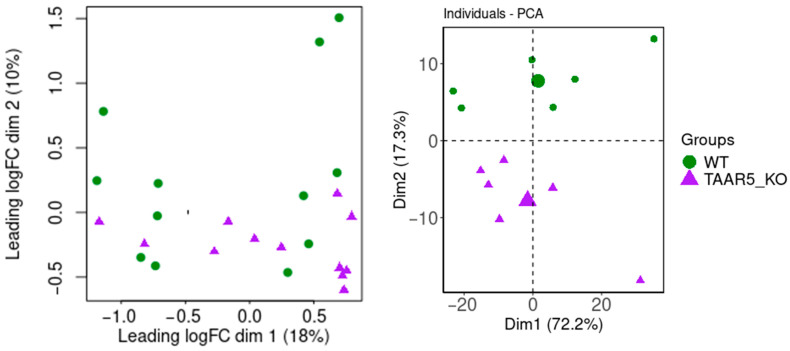
Multidimensional scaling (MDS) based on the log-fold changes between each pair of RNA samples of the top 500 expressed genes (**a**) and principal component analysis (PCA) plot of the top 500 expressed genes (**b**) for striatum RNA sequencing in control samples vs. TAAR5-KO samples.

To estimate similarities and differences in striatal expression profiles among WT and TAAR5-KO mice, we applied two dimensionality reduction approaches, i.e., MDS (Figure 1a) and PCA (Figure 1b). Briefly, MDS is more focused on the objects’ similarity, so similar objects in the *n*-dimensional space will be close together on the two-dimensional plot, while PCA seeks to maximize explained variance and uses a covariance/correlation matrix to analyze the correlation between data points and variables.

An MDS plot (Figure 1a) based on the log-fold changes between each pair of samples (Figure 1a) demonstrates that biological replicates from the same group (i.e., WT or TAAR5-KO) are clustered together rather than strewn. The plot represents differences in the striatal transcriptomic profile of these two groups of mice. Nevertheless, some samples of TAAR5-KO animals are more similar to WT samples. So, the first (x) dimension of the MSD plot, which describes 28% of the transcriptomic variance between samples, separates only half of the TAAR5-KO samples from the WT.

PCA of the same data provides clearer separation between TAAR5-KO and WT mice striatal expression profiles. The first two principal components (PC1 and PC2) describe 89.5% of the total variance in the data. PC2, which contains 17.3% of variability, however, can differentiate the two groups (Figure 1b).

### 3.2. Differentially Expressed Genes and Pathways in TAAR5-KO and WT Mice Striatum

**Figure 2:**
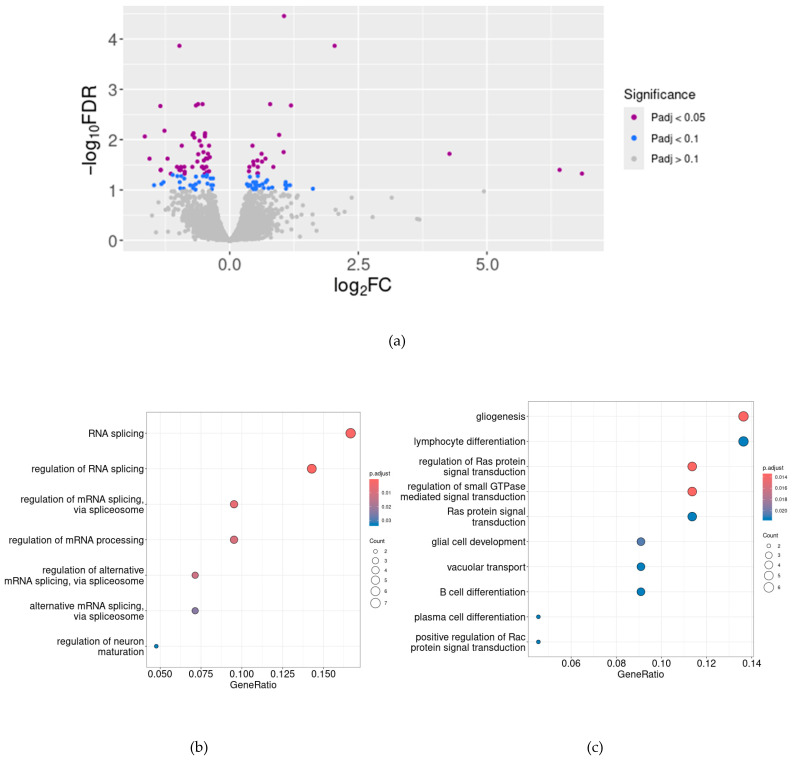
Differential expression analysis in the striatum of TAAR5-KO and WT mice. The volcano plot (**a**) shows the fold change (*x*-axis) versus the *P*_adj_ (*y*-axis). The significance (*P*_adj_) and the fold-change are converted to −Log_10_(*P*_adj_) and Log_2_(fold-change), respectively. The genes increased in the striatum of TAAR5-KO mice are represented in the upper-right part of the volcano, i.e., have log_2_ FC >  0, and genes that were decreased in the striatum of TAAR5-KO mice are in the upper-left part of the plot. The dot plots represent the Gene Ontology (GO) biological process terms’ enrichment in the cluster of genes, which are higher expressed in TAAR5-KO mice (**b**) or WT (top 10 terms are represented, (**c**)).

After filtering genes with low expression levels, as described above, 13,053 features were included in the differential expression analysis. Volcano plots represent the differential gene expression analysis in TAAR5-KO and WT mice (Figure 2a). Considering the mild differences in the cognitive function of TAAR5-KO and WT mice [8] and consequently applying the GO enrichment test for data interpretation, we used the tolerable cut-off value *P*_adj_ = 0.1. We identified 122 DEGs, 50 of which were upregulated in TAAR5-KO and 72 of which were more expressed in WT mice, i.e., only 0.1% of genes expressed in the striatum demonstrate a statistically significant change in expression level in response to TAAR5 knockout.

To understand the role of the identified 122 DEGs in biological processes, we performed GO term enrichment analysis. We obtained seven significantly enriched GO terms in the cluster of genes upregulated in the striatum of TAAR5-KO mice. These terms suggest the activation of transcription and post-transcriptional mRNA modification processes in TAAR5-KO mice. Moreover, the top upregulated genes include several genes involved in central nervous system function, which is described in detail in Table 1.

The GO terms enrichment analysis of genes, which are highly expressed in WT compared to TAAR5-KO mice, demonstrates that TAAR5 gene deletion suppresses genes involved in cell differentiation, especially in gliogenesis and activation of Ras signal transduction (in particular, *Camk2a*, *Myoc*, *Adgrd1*, and *Dock2*). Several genes listed in Table 1 are involved in nervous system development and functioning in the striatum and also are suppressed in the striatum of TAAR5-KO mice.

Considering the heterogeneity of study groups, which include both male and female animals, we estimated the sex-related differences in the expression levels of genes that are differently expressed between TAAR5-KO mice and WT. Most of these genes showed similar expression levels in male and female mice regardless of the genotype. Only TAAR5-KO-associated non-coding RNA A330076H08Rik had slightly higher expression in TAAR5-KO females compared to male knockout. In addition, TAAR5-KO females exhibit high expression of Glutathione S-transferase Mu 1 gene mRNA, which is downregulated in the TAAR5-KO mice. In WT mice, predicted genes Gm7292 and Gm14681 and pseudogene Gm15772 were identified only in females, but their mRNAs are at least completely lost in TAAR5-KO or WT male striatum (*P*_adj_ < 0.05 for all genes listed above).

### 3.3. Gene Coexpression Network Analysis Revealed Association of TAAR5 Gene Knockout in Mice with Neuron Arborization and Synaptogenesis

**Figure 3:**
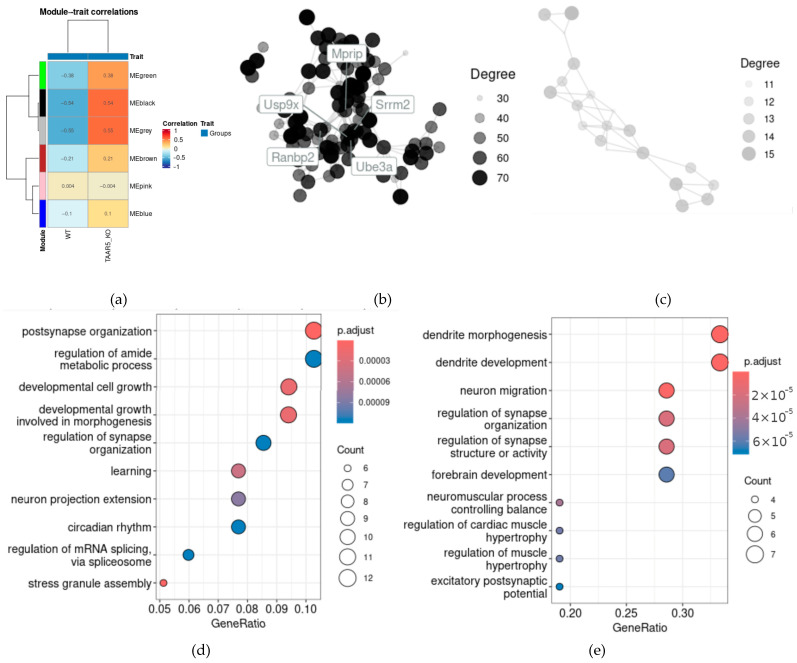
Gene coexpression network alteration in the striatum of TAAR5-KO mice. (**a**) Heatmap of the module–trait relationships with corresponding correlation coefficients; (**b**) coexpression network, represented correlations between the most coexpressed genes in the “black” cluster (r > 0.9); the list of nodes (genes) is listed in the Supplementary Table S1; (**c**) coexpression network, represented correlations between the most coexpressed genes in the “gray” cluster; the list of nodes (genes) is listed in the Supplementary Table S2; the dot plots representing the Gene Ontology (GO) biological process terms’ enrichment in the “black” (**d**) and gray (**e**) modules, respectively.

By comparing the TAAR5-KO striatal transcriptome with WT, WGCNA identified six coexpressed gene modules (Figure 3a). The module “black” comprises 122 genes, 119 of which were correlated with at least 1 other gene in the module with r > 0.9 and showed the strongest positive association with the TAAR5-KO genotype, as represented in Figure 3a. The TAAR5-KO genotype is also associated with the second module of coexpressed genes (i.e., the “gray” module) with a correlation coefficient of 0.54. This “gray” module includes only 23 genes, and 21 of them showed a correlation with at least 1 other gene in the module, with a correlation coefficient greater than 0.9.

The “black” module correlation network comprises 1618 edges whose topology is represented in Figure 3b. Also, we identified 12 hub genes, which may be principal to the regulation of the identified network. These genes include Etnk1 and Bltp1, which are involved in phosphatidylethanolamine metabolism; Son and Srrm2, which participate in splicing; Ube3a and Usp9x, which regulate ubiquitin-dependent protein degradation; and Prrc2c, Serbp1, Tcaf1, Atp6v1d, Ranbp2, Bltp1, and Mprip. All of these genes except Bltp1 are enriched in developing neurons and oligodendrocytes. Notably [51], ten of these hub genes (except Bltp11 and Mprip) are overrepresented in adult neurons in line with the BRETIGEA (BRain cEll Type specIfic Gene Expression Analysis) gene signatures [52]. Such associations may mirror the previously identified activity of neurodevelopment and neuronal activity in TAAR5-KO striatum [10]. The “gray” module coexpression network is less dense and includes 85 edges. Figure 3c represents the topology of the “gray” module coexpression network. There were no hub genes identified in this network.

According to the GO enrichment test results, both identified modules contain genes that are involved in striatal neuronal interaction, including genes that regulate cell growth, morphogenesis, and synaptogenesis (Figure 3d,e).

### 3.4. Transcriptomic Deconvolution Reveals Change Both in Neurons and Glial Cell Content in the Striatum of TAAR5-KO Mice

**Figure 4:**
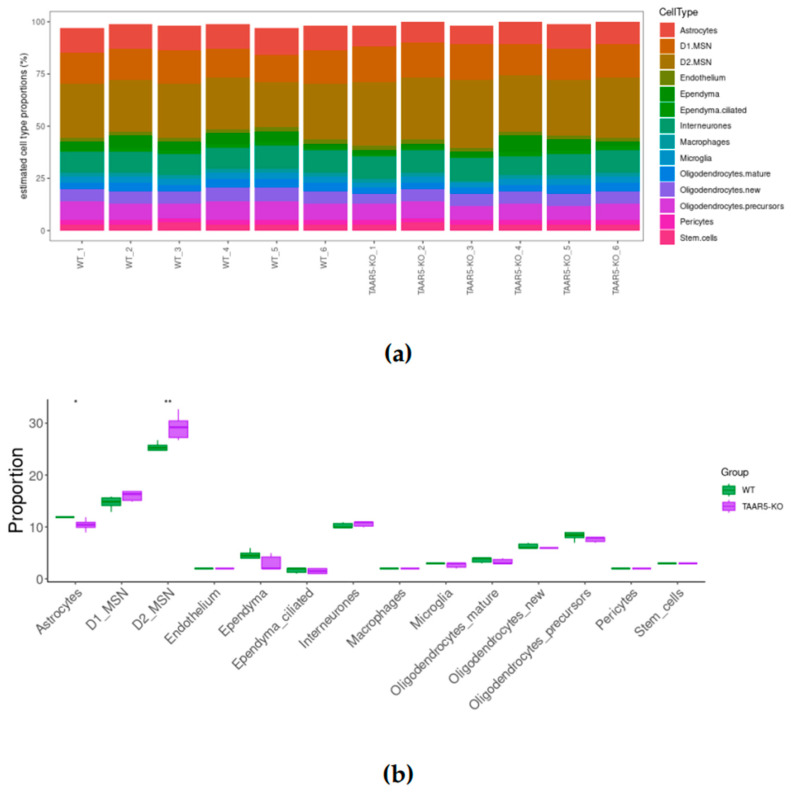
Estimation of cell-type proportions in TAAR5-KO and WT striatum by bulk RNA sequencing deconvolution. (**a**) A bar plot illustrating the total proportions of cell types for each sample. Each bar represents an individual sample, *y*-axis shows the estimated proportion of each of the five cell types. Samples were ranged automatically by similarity; V7–V12 are TAAR5-KO samples, and V13–V18 are WT samples. (**b**) The box plot represents the relative abundances of cell fractions in the striatum of TAAR5-KO and WT mice. *—*p* value < 0.05, **—*p* value < 0.01.

We carried out reference-based bulk RNA sequencing deconvolution to identify the cell composition of the studied samples. The TAAR5-KO and WT RNA sequencing data (Figure 4a) allowed us to identify all cell groups included in the reference mouse striatum profile. The proportions of different cell types are congruent with those described previously [53]. Summarized proportions of the cell types are close to 100%.

When comparing cell fractions subsets between TAAR5-KO and WT samples, the striatum from TAAR5-KO mice demonstrated a significantly higher relative abundance of D2 dopamine receptor-expressing GABAergic medium spiny neurons (*p* = 0.0076) and slightly lower relative abundance of astrocytes (*p* = 0.01) (Figure 4b).

Thus, we compared the expression profiles among WT and TAAR5-KO mice by different statistical approaches and identified that, despite the mild differences induced by TAAR5 knockout, several differentially expressed genes are involved in neuronal function, nervous system development, maturation, and performance.

## 4. Discussion

Previously, TAAR5 expression was identified in various limbic brain areas, including striatum [7]. TAAR5 elimination in these animals Electrophysiologic and behavioral tests show the decrease in delta power spectral density, identified in the striatum of TAAR5-KO mice suggested to be associated with motivation, attention, and concentration. These cognitive functions are associated with motor coordination and balance ability, which improvement was identified in TAAR5-KO mice compared to their WT littermates [8,9]. Additionally, TAAR5-KO mice demonstrate less anxiety- and depressive-like behavior and improved cognitive functions, given the fewer errors in the timing tasks [5,6].

In the present report, we attempted to present a more detailed description of striatal alterations associated with TAAR5 gene knockout in mice.

Our study revealed slight but significant shifts in the striatal expression profile in TAAR5-KO mice. These differences may involve not only the regulation of the expression of some single genes but also can restructure of the expression of gene modules and, possibly, modify striatal cell composition. The identified changes probably represent the molecular background of behavioral features of TAAR5-KO mice, which were described previously [5]. The applied methods demonstrate that the patterns of most expressed genes in TAAR5-KO and WT groups distinguish these two genotypes despite the low number of DEGs. The identified DEGs, which discriminate TAAR5-KO and WT mice striatal expression profiles, are associated with RNA splicing, Ras signaling, cell differentiation, or striatum functioning and dopaminergic regulation.

The identified upregulation of *Lrrk2* expression, a member of the leucine-rich repeat kinase family and mutations that have been associated with Parkinson’s disease, may be relevant in the context of previously described enhancement of dopaminergic neurotransmission in the TAAR5-KO mouse striatum. The product of this gene is involved in vesicle trafficking and cytoskeleton regulation, including interactions with microtubules or actin, regulation of endolysosomal system activity, and dopaminergic neurotransmission in the striatum [36]. In particular, it regulates D1 receptor internalization and D2 receptor trafficking from the Golgi complex to the cell membrane [37]. The dysfunction of Lrrk2 is associated with impaired striatal synaptogenesis and the control of ambulatory and fine movement [36].

Splicing is a basic cellular process required for gene expression. Both the reaction to external circumstances and pathologic conditions may relate to the alteration of splicing in the striatum. Previously, the alterations of spliceosome component expression in the striatum were identified in different contexts, including the models of disabling diseases, such as autism spectrum disorder [54] and neurodegeneration in Huntington’s disease [55], or after the repeated social conflicts in experimental conditions [56].

Ras-ERK signal transduction cascade, whose components are downregulated in TAAR5-KO mice, is involved in the MSN synaptic plasticity; however, its activation in the striatum may be associated with compulsive behavior [57], drug addiction [58], neurodegeneration, or L-DOPA-induced dyskinesia [59,60]. ERK is involved in the rewarding effect of substances with abuse potential [61] and activates after such compound administration [62]. However, the ameliorative effect of mood stabilizers like valproate or endogenic neurotrophic factors on neurodevelopment [63,64,65], as well as corticostriatal synaptic plasticity, is also mediated by ERK. Its activation in the dorsal striatum is necessary for goal-directed behaviour. Meanwhile, in the ventral striatum, ERK is involved in the reward and motivation [66]. Following the complex role and spatial organization of RAS-ERK signaling in the striatum, further studies are necessary to identify the result of a slight decrease in RAS-ERK signaling component expression in the TAAR5-KO mice. So, the functional significance of Ras-signal transduction and splicing alterations in TAAR5-KO mice striatum needs further investigation.

Also, we identified the downregulation of several genes involved in gliogenesis and glial cell differentiation in TAAR5-KO mice, accompanied by the upregulation of genes associated with neuronal differentiation, axonogenesis, and synaptogenesis. In line with these results, we identified the increased proportion of MSN in the striatum of TAAR5-KO mice by deconvolution of our bulk RNA sequencing. MSN is considered a target for dopamine from substantia nigra pars compacta, and these neurons are the major population of striatal neurons, which is characterized by dense dopamine innervation and high levels of dopamine D1 (D1R) and D2 (D2R) receptor expression [58]. Both MSN populations are more represented in TAAR5-KO mice compared to WT; however, only D2 MSN overrepresentation reaches a statistically significant level. It can be noted that the change in the cell composition of TAAR5-KO mice striatum is accompanied by the upregulation of Trank1 gene expression. This gene was previously identified as a potential marker of both MSN subtypes. On the other hand, despite the biological function of Trank1 in the brain remaining unknown, it is suggested to be involved in immune-related signal regulation [40], synaptic plasticity, and axon guidance [38], and its depletion is associated with anxiety-like behavior [34,67]. So, Trank1 upregulation in TAAR5-KO striatum may mirror cell composition or some compensation of the anxiolytic effect of TAAR5 knockout.

Astrocytes participate in dopaminergic neurotransmission in the striatum [53], express all classes of dopamine receptors [54,55,56], are involved in the striatal dopamine metabolism [57], play a crucial role in the regulation of synaptic strength [68], astrocytes modulate the behavioral output of the mesolimbic dopamine system, specifically reward-related and motivated behaviors [69]. The present study did not make it possible to identify the reason for the slight astrocyte number reduction in knockout mice. However, it may be assumed that the decline of astrocyte proportion in the striatum of TAAR5-KO mice may be related to the changes in dopamine neurotransmission and metabolism. However, further studies of the role of TAARs in the functioning of astrocytes and the influence of the trace amine system and its receptors on glia are extremely important.

Taking into account the weak degree of identified TAAR5 depletion-related alterations described above, we additionally analyzed the changes in gene coexpression networks between TAAR5-KO and WT mice. These results demonstrated the activation of genes that are involved in morphogenesis, especially in neurogenesis, synaptogenesis, and neural differentiation.

Intriguingly, recent evidence suggests that adult neurogenesis may occur not only in subventricular and subgranular zones of the brain but also in several other zones, including the striatum [53]. The identified complex upregulation of genes involved in neuronal network morphogenesis may mirror previously described activation of adult neurogenesis in TAAR5-KO mice [54]. The identified associations also are consistent with previously identified improvement of cognitive functions in TAAR5 knockout mice [13].

Striatal synaptic plasticity is suggested as a potential mechanism for the acquisition and maintenance of controlling timing behavior [70]. Previously, TAAR5-KO mice demonstrated higher performance in temporal decision-making tasks compared to WT littermates. The synaptic plasticity of striatal neurons is also significant for motor coordination and balance, as it was identified in the neurodegenerative disease models [71,72,73,74]. It may be speculated that the activation of genes involved in synaptogenesis and synapse organization, identified in the present study, is the molecular basis of complex motor coordination improvement in TAAR5-KO mice along with TAAR5-KO effect on the cerebellum [8].

The main strength of the present study is the application of RNA sequencing for the estimation of TAAR5 knockout in the gene expression profile, as this method is precise and has high sensitivity, which allows for the detection of low-abundance transcripts and mild differences in gene expression levels between study groups. However, our study has some limitations. A considerable proportion of identified alterations are associated with the neuron’s growth and development, or synaptogenesis. Thus, the animals’ age (10 months) may mitigate the impact of TAAR5 gene knockout on these processes. It will be necessary to further confirm these identified relationships in younger animals, demonstrating more intensive neuron arborization. Likewise, our conclusions are based on the study of three male and three female mice in each group (TAAR5-KO and WT), which might limit the generalizability of the findings and reduce the statistical power of detecting subtle changes in gene expression. Another significant limitation is the selected false discovery rate cut-off value. The genes for which *P*_adj_ < 0.1 were considered significantly differentially expressed in TAAR5-KO mice in this study. To overcome this limitation, we analyzed the identified gene clusters using the GO enrichment method, which identifies genes associated with common functions. Despite these limitations, the identified features of the striatal transcriptome in TAAR5-KO mice are consistent with current findings and can be explained in light of previously published results. Also, there are limitations associated with the generalizability of findings from a mouse model to human conditions that can be of a fundamental nature. For example, species-related pharmacological differences were previously identified when human and mouse TAAR1 ligand specificity was estimated [75].

The present findings demonstrate that the elimination of TAAR5 in mice may cause both expression pattern changes and cell composition alterations in the striatum. Additionally, WGCNA allows to reveal the pronounced differences in the expression of genes involved in the neurogenesis and synaptogenesis between TAAR5-KO mice and their WT littermates, and the cell type deconvolution analysis identified the higher relative abundance of D2 medium spiny neurons in the striatum These findings extend previous the results of previous studies that show activation of adult neurogenesis and cognitive functions and motor coordination in TAAR5-KO mice. Since the TAAR5 knockout effects, among others, are associated with the decline of anxiety-like and depressive-like behavior in mice, it is considered a prospective target for neuropsychiatric disorders pharmacotherapy. Further studies are necessary to identify the impact of TAAR5 knockout or inactivation on the behavior and cerebral functions, including the investigation of the molecular background of the identified phenotypic changes.

## 5. Conclusions

TAAR5 is the second of the most studied trace amine-associated receptors after TAAR1, which is suggested to recognize tertiary amines like trimethylamine. Previously, its knockout in mice demonstrated the enhancing effect on neurogenesis and dopaminergic neurotransmission. To estimate the role of the TAAR5 gene in the striatum, we performed large-scale transcriptome profiling in the striatum using RNAseq in TAAR5-KO mice and their wild-type littermates. Our results confirm the TAAR5 significance for striatal morphogenesis and functioning. Estimating differential gene expression revealed the impact of TAAR5 gene depletion on the genes involved in dopaminergic signaling regulation and genes involved in mRNA transcription and maturation. Meanwhile, genes involved in mRNA transcription and maturation are also higher expressed in TAAR5-KO mice striatum. Genes involved in gliogenesis and genes of Ras-ERK axis components are downregulated in the striatum TAAR5-KO mice. The gene coexpression network analysis revealed associating TAAR5 knockout with cell growth, morphogenesis, and synaptogenesis. In accordance with these data, the deconvolution analysis revealed that TAAR5-KO striatum samples contain more D2 dopamine receptor-expressing MSNs than in wild-type mice and a lower relative abundance of astrocytes.

However, the observed effect of TAAR5 gene knockout on cell type proportions in mouse striatum points to the need for a more detailed investigation of the role of TAAR5 in glial processes in middle-aged subjects. Also, considering the effect of TAAR5 gene knockout on the cell type proportions in mouse striatum points to the need for a more detailed investigation of the role of TAAR5 in glial processes in middle-aged subjects’ composition of a 10-month-old mouse, it may be interesting from the point of view of striatum formation of different developmental stages and its aging. Further studies in younger animals and comparison of sequencing data from other brain regions associated with the dopaminergic system and others will be essential to understanding the functions of TAAR5 and the processes in which they participate. This is especially important in considering TAAR5 as a prospective target for therapy of neuropsychiatric disorders.

## Figures and Tables

**Table 1:** The genes involved in central nervous system function which are differentially expressed in TAAR5-KO striatum.

Gene	Function in the Nervous System and Dopaminergic Neurotransmission
Upregulated in TAAR5-KO	
*Lrrk2*	A member of the leucine-rich repeat kinase family involved in vesicle trafficking, cytoskeleton regulation—including interactions with microtubules or actin—and regulation of endolysosomal system activity [35] is suggested to also be involved in dopaminergic signaling regulation in the striatum [36,37], neurodegeneration, and Parkinson’s disease pathogenesis [38].
*Trank1*	The bipolar disorder risk gene [39], which is highly expressed in the striatal neurons [34], is suggested to be involved in immune-related signal regulation [40], synaptic plasticity, and axon guidance [38].
*Gpr155*	The Orphan GPCR is commonly expressed in the striatum and hippocampus and takes part in cognition and limbic systems functioning [41].
*Calb1*	Expressed in the substantia nigra dopamine neurons and is involved in the reward response [42].
*Mir124a-1hg*	Involved in axonogenesis and synaptogenesis [43].
*Cntnap3*	
*Lrtm1*	Suggested to be a surface marker of dopamine neuron progenitors [44].
Downregulated in TAAR5-KO	
*Tunar*	Negative regulators of neural differentiation [45,46].
*Disp3*	
*Grin2c*	Glqun2C subunit of the NMDA receptor, which modulates dopamine release in the striatum [47].
*Olig1*	Oligodendrocyte transcription factor 1 is involved in oligodendrocyte development and, especially, in remyelination in the adult brain [48].
*Camk2d*	The gene of calcium/calmodulin-dependent protein kinase II (CaMKII) is principal for striatum-associated cognitive function and striatal synaptic transmission [49] and functions like an excitability rheostat of striatal MSNs by coordinating excitatory synaptic drive and the resulting depolarization response [50].

## Data Availability

The GEO (Gene Expression Omnibus) accession number for the RNA sequencing data reported in this paper is GSE276664. Accessed on 8 September 2024.

## Supplementary Materials

The following supporting information can be downloaded at https://www.mdpi.com/article/10.3390/cells13221910/s1. Table S1: “Black” coexpression striatal transcriptome module related to the TAAR5-KO genotype; Table S2: “Grey” striatal coexpression transcriptome module related to the TAAR5-KO genotype.
