# Supplementary material for: Knocking Out TAAR5: A Pathway to Enhanced Neurogenesis and Dopamine Signaling in the Striatum
Source: Cells. 2024 Nov 19;13(22):1910. doi: 10.3390/cells13221910 (PMC11592834; doi:10.3390/cells13221910)
Supplement: Supplementary file 1 [file cells-13-01910-s001.zip › cells-3248862-supplementary.pdf]

SUPPLEMENTARY

Supplementary Table S1.

"Black" co-expression striatal transcriptome module related with TAAR5-KO genotype

Gene Symbol	Gene name
Dst	dystonin
Acsl3	acyl-CoA synthetase long-chain family member 3
Unc80	unc-80, NALCN activator
Ncl	nucleolin
Prrc2c	proline-rich coiled-coil 2C
Akt3	thymoma viral proto-oncogene 3
Hnrnpu	heterogeneous nuclear ribonucleoprotein U
Cdc42bpa	CDC42 binding protein kinase alpha
Gad1	glutamate decarboxylase 1
Meis2	Meis homeobox 2
Bltp1	bridge-like lipid transfer protein family member 1
Cacna1e	calcium channel, voltage-dependent, R type, alpha 1E subunit
Norad	non-coding RNA activated by DNA damage
Snhg11	small nucleolar RNA host gene 11
Nckap1	NCK-associated protein 1
Caprin1	cell cycle associated protein 1
Nbea	neurobeachin
Csde1	cold shock domain containing E1, RNA binding
Ube2d3	ubiquitin-conjugating enzyme E2D 3
Trim44	tripartite motif-containing 44
Pcdh10	protocadherin 10
Gria2	glutamate receptor, ionotropic, AMPA2 (alpha 2)
Gpr88	G-protein coupled receptor 88
Ptprd	protein tyrosine phosphatase receptor type D
Lrrc7	leucine rich repeat containing 7
Penk	preproenkephalin
Prkacb	protein kinase, cAMP dependent, catalytic, beta
Negr1	neuronal growth regulator 1
Ppp1cb	protein phosphatase 1 catalytic subunit beta
Ttll7	tubulin tyrosine ligase-like family, member 7
Zfp644	zinc finger protein 644
Macf1	microtubule-actin crosslinking factor 1

Tcaf1	TRPM8 channel-associated factor 1
Hnrnpa2b1	heterogeneous nuclear ribonucleoprotein A2/B1
G3bp2	G3BP stress granule assembly factor 2
Serbp1	serpine1 mRNA binding protein 1
Etnk1	ethanolamine kinase 1
Gabrb3	GABRB3, gamma-aminobutyric acid type A receptor subunit beta 3
Ube3a	ubiquitin protein ligase E3A
Ipw	imprinted gene in the Prader-Willi syndrome region
Akap9	A kinase anchor protein 9
Grm5	glutamate receptor, metabotropic 5
Bcas1	brain enriched myelin associated protein 1
Spock3	sparc/osteonectin, cwcv and kazal-like domains proteoglycan 3
Ptn	pleiotrophin
Fam168a	family with sequence similarity 168, member A
Calr	calreticulin
Fat3	FAT atypical cadherin 3
Sesn3	sestrin 3
Aplp2	amyloid beta precursor-like protein 2
Arhgef12	Rho guanine nucleotide exchange factor 12
Ntrk3	neurotrophic tyrosine kinase, receptor, type 3
Septin7	septin 7
Myo9a	myosin IXa
Herc1	HECT and RLD domain containing E3 ubiquitin protein ligase family member 1
Ddx6	DEAD-box helicase 6
Arpp19	cAMP-regulated phosphoprotein 19
Syne1	spectrin repeat containing, nuclear envelope 1
Serinc1	serine incorporator 1
Tmem30a	transmembrane protein 30A
Ranbp2	RAN binding protein 2
Plcb1	phospholipase C, beta 1
Ubr3	ubiquitin protein ligase E3 component n-recognin 3
Eps15	epidermal growth factor receptor pathway substrate 15
Usp34	ubiquitin specific peptidase 34
Rtn4	reticulon 4
Aff4	AF4/FMR2 family, member 4
Anks1b	ankyrin repeat and sterile alpha motif domain containing 1B
Nsg2	neuron specific gene family member 2

Mprip	myosin phosphatase Rho interacting protein
Taok1	TAO kinase 1
Atp2b1	ATPase, Ca++ transporting, plasma membrane 1
Cyld	CYLD lysine 63 deubiquitinase
Spag9	sperm associated antigen 9
Ddx5	DEAD box helicase 5
Spock2	sparc/osteonectin, cwcv and kazal-like domains proteoglycan 2
Dgkb	diacylglycerol kinase, beta
Arhgap5	Rho GTPase activating protein 5
Atp6v1d	ATPase, H+ transporting, lysosomal V1 subunit D
Hsp90aa1	heat shock protein 90, alpha (cytosolic), class A member 1
Myt1l	myelin transcription factor 1-like
Eif5	eukaryotic translation initiation factor 5
Prkar1a	protein kinase, cAMP dependent regulatory, type I, alpha
Nrxn3	neurexin III
Slmap	sarcolemma associated protein
Fzd3	frizzled class receptor 3
Akap11	A kinase anchor protein 11
Nrsn1	neurensin 1
Rims2	regulating synaptic membrane exocytosis 2
Mycbp2	MYC binding protein 2, E3 ubiquitin protein ligase
Mbnl2	muscleblind like splicing factor 2
Son	Son DNA binding protein
Ttc3	tetratricopeptide repeat domain 3
Tulp4	tubby like protein 4
Srrm2	serine/arginine repetitive matrix 2
App	amyloid beta precursor protein
Hsp90ab1	heat shock protein 90 alpha (cytosolic), class B member 1
Enpp5	ectonucleotide pyrophosphatase/phosphodiesterase 5
Pde10a	phosphodiesterase 10A
Meg3	maternally expressed 3
Calm2	calmodulin 2
Ankrd12	ankyrin repeat domain 12
Pura	purine rich element binding protein A
Smarca2	SWI/SNF related, matrix associated, actin dependent regulator of chromatin, subfamily a, member 2
Usp9x	ubiquitin specific peptidase 9, X chromosome

Rgs7bp	regulator of G-protein signalling 7 binding protein
Kcnq3	potassium voltage-gated channel, subfamily Q, member 3
Mobp	myelin-associated oligodendrocytic basic protein
Malat1	metastasis associated lung adenocarcinoma transcript 1 (non-coding RNA)
Kif5b	kinesin family member 5B
Atrx	ATRX, chromatin remodeler
Prepl	prolyl endopeptidase-like
Gnal	guanine nucleotide binding protein, alpha stimulating, olfactory type
Ddx3x	DEAD box helicase 3, X-linked
Arhgef9	CDC42 guanine nucleotide exchange factor 9
Plp1	proteolipid protein (myelin) 1
Tro	trophinin
Huwe1	HECT, UBA and WWE domain containing 1
Cnksr2	connector enhancer of kinase suppressor of Ras 2

Supplementary Table S2.

"Grey" striatal co-expression transcriptome module related with TAAR5-KO genotype

Gene Symbol	Gene name
Spred1	sprouty protein with EVH-1 domain 1, related sequence
Ano3	anoctamin 3
Caln1	calneuron 1
Ppp3ca	protein phosphatase 3, catalytic subunit, alpha isoform
Mef2a	myocyte enhancer factor 2A
Ppp1r9a	protein phosphatase 1, regulatory subunit 9A
Camk2b	calcium/calmodulin-dependent protein kinase II, beta
Myh10	myosin, heavy polypeptide 10, non-muscle
Hspa8	heat shock protein 8
Arpp21	cyclic AMP-regulated phosphoprotein, 21
Rasl10b	RAS-like, family 10, member B
Bcl11b	B cell leukemia/lymphoma 11B
Phactr1	phosphatase and actin regulator 1
Dgkh	diacylglycerol kinase, eta
Mrtfb	myocardin related transcription factor B
Adcy5	adenylate cyclase 5
Gsk3b	glycogen synthase kinase 3 beta

Tomm70a	translocase of outer mitochondrial membrane 70A
Pafah1b1	platelet-activating factor acetylhydrolase, isoform 1b, subunit 1
Pum2	pumilio RNA-binding family member 2
Neto1	neuropilin (NRP) and tolloid (TLL)-like 1
